# The Young Stethoscopist, &c.

**Published:** 1848

**Authors:** 


### ARTICLE IV.

*The Young Stethoscopist, or the Students' Aid to Auscultation.* By HENRY S. BOWDITCH, M.D., one of the Physicians to the Mass. Gen. Hospital. Second Edition. New York: S. S. & W. Wood, 1848. (From the Publishers—for sale by Joseph Keene, Jr., Chicago.)

This is a convenient little volume, and the call for a second edition seems conclusive of its meeting with favor at the hands of the profession. The subject of which it treats is one too much neglected by the profession generally, as auscultation both mediate and direct, furnishes, when rightly understood, one of the most important and clear means of detecting disease. The author has faithfully laid down the principles that should govern in its application, which will afford a correct guide to practice the only means of acquiring the tact and discrimination of sounds necessary to render it useful. [E.

